# Delayed Onset Iliopsoas Tendonitis With Intramuscular Hematoma Following Total Hip Arthroplasty

**DOI:** 10.1016/j.artd.2024.101589

**Published:** 2024-12-09

**Authors:** Kevin S. Orton, Jonathan M. Stern, Natalia Cruz-Ossa, Freddy A. Hung, Antonio Fernandez-Perez, Jean Jose, Victor Hugo Hernandez

**Affiliations:** a Department of Orthopaedics, University of Miami Hospital, Miami, FL, USA; b Department of Radiology, University of Miami Hospital, Miami, FL, USA

**Keywords:** Iliopsoas tendonitis, Intramuscular hematoma, Total hip arthroplasty, Revision total hip arthroplasty, Case report

## Abstract

Iliopsoas tendonitis following total hip arthroplasty (THA) can be challenging to diagnose due to the many causes of postoperative groin pain. This case involves a 66-year-old female with right-sided hip and groin pain and a palpable mass, 3 years post-THA. Initial recovery was unremarkable until the sudden onset of symptoms after exercise. The patient presented with a palpable mass on the groin area and pain and underwent several consults with general surgery and orthopaedics. A series of unremarkable radiogram, biopsies, and negative infectious workup prompted revision surgery, which included acetabular component revision and iliopsoas tenotomy. The patient experienced significant pain relief and improved mobility postrevision. This case highlights a complication of THA and the complexity of diagnosing and treating iliopsoas tendonitis post-THA.

## Introduction

Diagnosing iliopsoas tendonitis following total hip arthroplasty (THA) can present a difficult challenge to the orthopaedic surgeon due to the numerous causes of postoperative groin pain after THA, with most pathologies presenting similarly. Iliopsoas tendonitis as a cause of groin pain following THA is well-established, with a reported incidence between 4% and 29% of patients after THA [1]. Iliopsoas tendonitis is most commonly associated with anterior scar tissue and an exposed acetabular component secondary to retroversion, lateralization, or oversizing [2]. Other causes include anterior osteophyte impingement, misplaced screws, cement extravasation, and leg length discrepancies [3].

Iliopsoas tendinitis typically occurs approximately 12-24 months after surgery, but can present any time between 2 and 96 months postoperatively [4]. Although iliopsoas tendinitis is a known cause of groin pain after THA, it is rarely associated with an intramuscular hematoma. The timing of symptom onset complicates the diagnosis and treatment of severe cases. We present an unusual case of iliopsoas tendonitis with associated hematoma, secondary mass effect causing pain, and functional decline 3 years after a previously well-functioning THA.

## Case history

**Figure 1:**
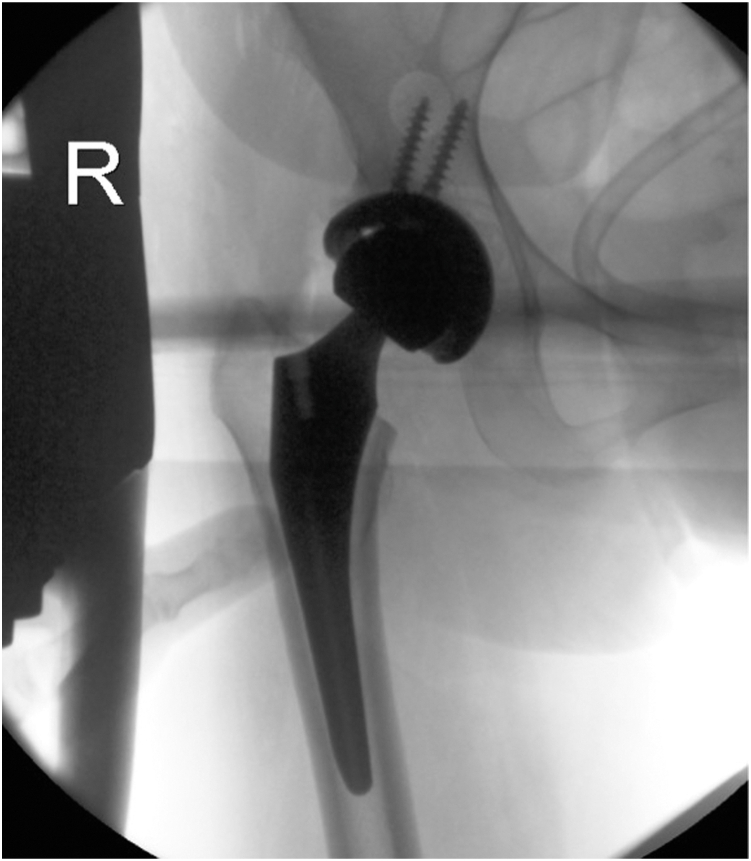
Intraoperative anterior posterior (AP) view of the right hip from the index total hip arthroplasty. Intraoperative radiogram demonstrating well-aligned femoral and acetabular components without signs of fracturing.

A 66-year-old female presented to the orthopaedic clinic in 2022 for a 3-year THA follow-up visit, complaining of a 4-month history of right-sided hip and groin pain with an associated palpable and visual anterior groin mass. She had previously undergone primary right THA via an anterior approach done by the senior author in June 2019 (Fig. 1). The patient had an unremarkable postoperative course for 2.5 years until the sudden onset of symptoms after exercise. Written informed consent from the patient was obtained to use her clinical history, radiogram, and intraoperative photos for presentation purposes.

**Figure 2:**
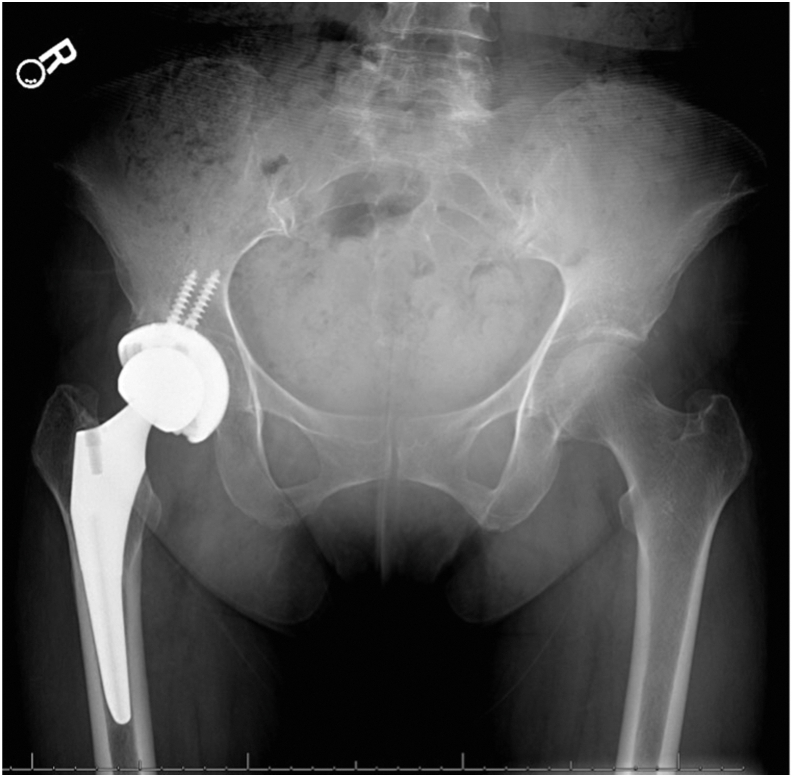
AP view of pelvis one-month postoperative from the index total hip arthroplasty. One-month postoperative radiogram demonstrating total hip arthroplasty with well-aligned components and without signs of loosening, fracture, or dislocation.

Her past medical history was significant for hyperlipidemia, osteoporosis, hypothyroidism, and left-sided parotid gland cancer, for which she underwent surgical resection in 1999, complicated by cranial nerve VII palsy requiring gold eyelid weight placement in 2014. Active medications included rosuvastatin, levothyroxine, and vitamin D supplements. The patient underwent right THA via a direct anterior approach at our institution in 2019 using a Stryker Trident (Stryker, Mahwah, NJ) 54 mm acetabular cup with 2 screws, Stryker Trident (Stryker, Mahwah, NJ) 36 MM liner, a #4 femoral stem, 36 mm ceramic femoral head, and a −5-mm neck. There were no intraoperative or immediate postoperative complications. At her 1 month postoperative visit, the patient reported right lower extremity discomfort with prolonged standing and ambulation but was otherwise pain-free. Her incision was well healed without erythema, tenderness, or swelling, and she ambulated with an antalgic gait without using an assistive device. Her right hip radiogram [anteroposterior (AP) and lateral] demonstrated a right THA with well-aligned hardware without signs of loosening, dislocation, or fracture (Fig. 2).

At her 6 months postoperative visit, the patient reported doing well without pain or difficulties. She could walk without assistance and perform all activities of daily living. Her active and passive right hip range of motion was pain-free, with hip flexion to 100 degrees. Her hip remained stable with abduction, adduction, and internal and external rotation. Repeated right hip AP and lateral radiogram were unremarkable.

In March 2020, the patient reported right hip soreness but continued to have a normal level of activity, with an unchanged physical exam or radiogram compared to prior visits. The patient was instructed to continue with daily living activities and physical therapy, with specific instructions on doing core exercises to lessen the pressure on her hip. The plan was to reassess progress in 1 year. However, she presented to her primary care provider in December 2021 with a 1.5-month history of intermittent right-sided groin pain that started suddenly after the patient was doing bodyweight squats. A physical exam revealed a soft, compressible, nontender inguinal bulge without overlying skin changes. The patient had no right lower extremity weakness and continued to have a full passive and active range of motion. She was referred to general surgery for a workup of a suspected inguinal hernia.

The patient underwent a right groin ultrasound in January 2022, which demonstrated a 3.2 cm avascular, isoechoic lesion in the right inguinal region, which was equivocal for a hernia. She was seen by a general surgeon, who was unable to appreciate an inguinal hernia on physical exam and ordered a computed tomography (CT) abdomen/pelvis without contrast. CT scan in March 2022 demonstrated a 3.8 × 3.9 × 0.8.6 cm intramuscular fluid collection within the right psoas muscle concerning for an intramuscular hematoma or seroma.

**Figure 3:**
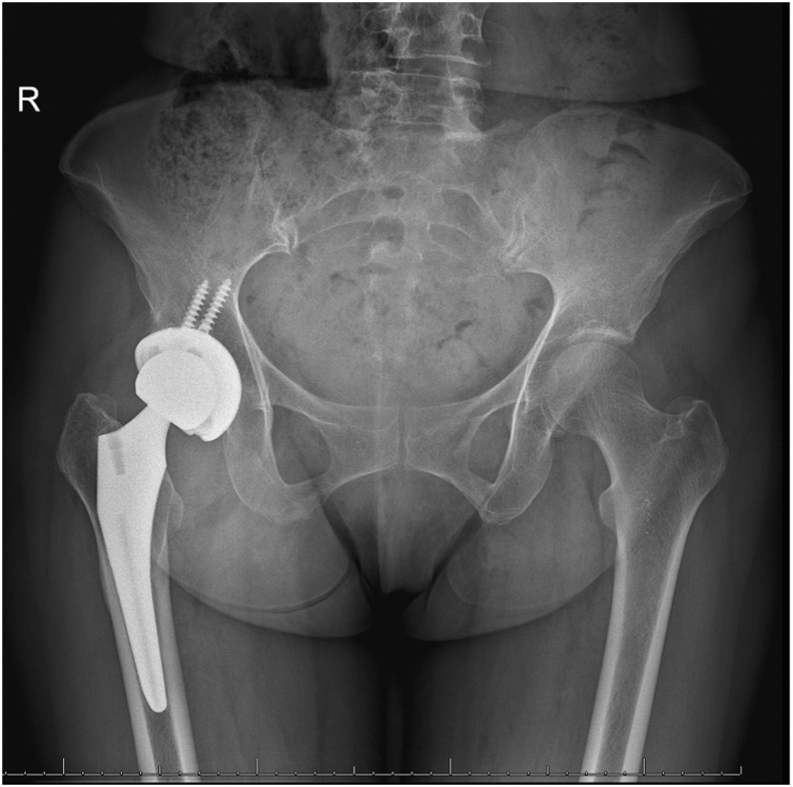
AP view of the pelvis approximately 3 years after index total hip arthroplasty. Radiogram demonstrates total hip arthroplasty in good alignment with no signs of loosening, fracture, or dislocation.

**Figure 4:**
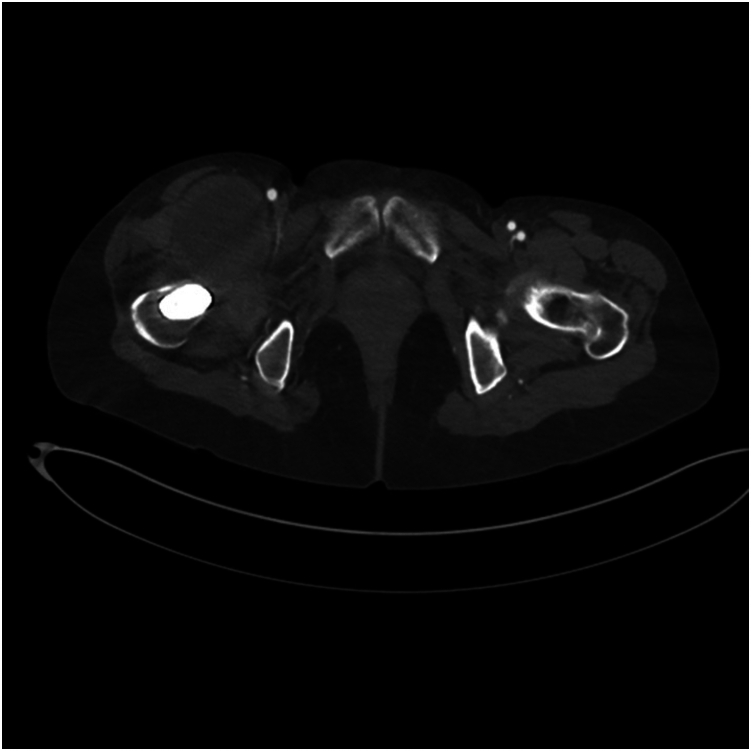
Axial view of right-sided iliopsoas hematoma. CT scan demonstrating a hypodense mass measuring up to 5.4 × 4.9 cm in cross-section and 9.7 cm in craniocaudal dimension.

**Figure 5:**
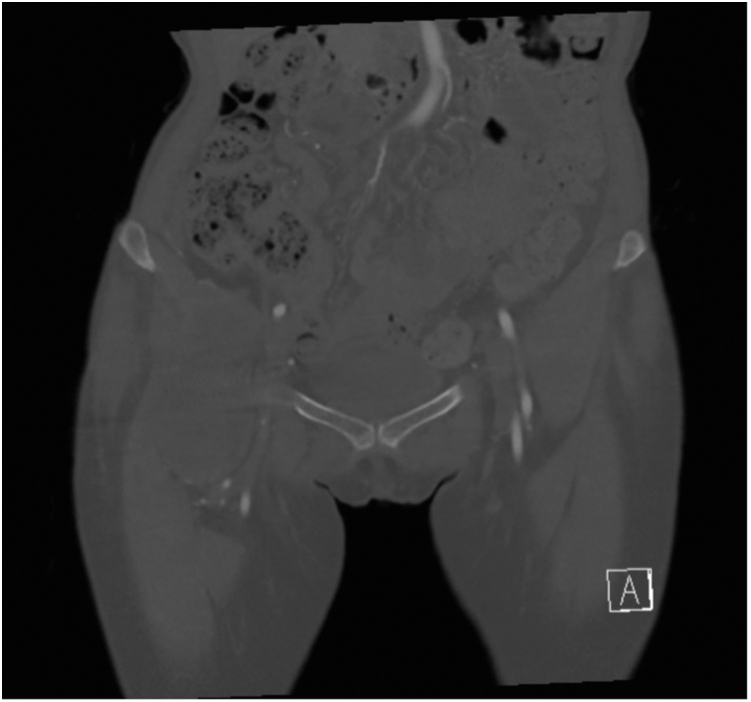
Coronal view of right-sided iliopsoas hematoma. CT scan demonstrating a hypodense mass measuring up to 5.4 × 4.9 cm in cross-section and 9.7 cm in craniocaudal dimension.

**Figure 6:**
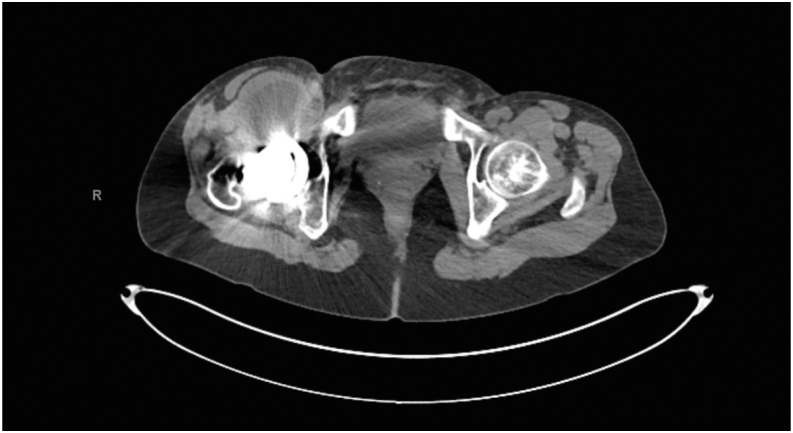
Axial view of the pelvis. CT scan demonstrating acetabular component overhang and impingement of the iliopsoas over the femoral head.

The patient presented to the orthopaedic clinic due to concerns about her right groin mass a week after her CT scan in March 2022. She reported pain with hip flexion and persistent swelling over the anterior hip region. Physical exam of the right lower extremity was significant for a palpable mass on the anterior aspect of the hip and limited hip flexion. Right hip AP and lateral radiogram demonstrated a well-aligned THA without component loosening, fracture, or dislocation (Fig. 3). Erythrocyte sedimentation rate and C-reactive protein (CRP) were within normal limits. The patient was referred for an ultrasound-guided aspiration, which demonstrated a partial-thickness iliopsoas tendon tear and a large hematoma extending through the capsule into the iliopsoas bursa. Approximately 110 cc of sanguineous fluid was aspirated and sent for cytology, which was negative for malignancy. To rule out infection, the aspirated fluid was sent for gram and acid-fast staining, bacterial and fungal cultures, and analysis using Synovasure Periprosthetic Joint Infection Panel (Zimmer Biomet, Warsaw, IN), which includes microbial detection, alpha-defensins, and CRP levels, which were all negative. A CT angiogram of the pelvis was obtained in May 2022 and was significant for a hematoma vs significant iliopsoas bursitis that measured up to 9.7 cm craniocaudally (Figure 4, Figure 5). Additionally, there was an anterior acetabular component overhang with iliopsoas impingement (Fig. 6). Differential diagnoses included muscle irritation, subacute venous bleeding from femoral head irritation, and pigmented villonodular synovitis. A magnetic resonance imaging(MRI) study would have been the next diagnostic step. However, her eye implant was incompatible. Instead, a right THA revision was recommended to visualize irritation of the iliopsoas caused by the implant, explore for active venous bleeding, and perform an intraoperative synovial biopsy to rule out pigmented villonodular synovitis.

**Figure 7:**
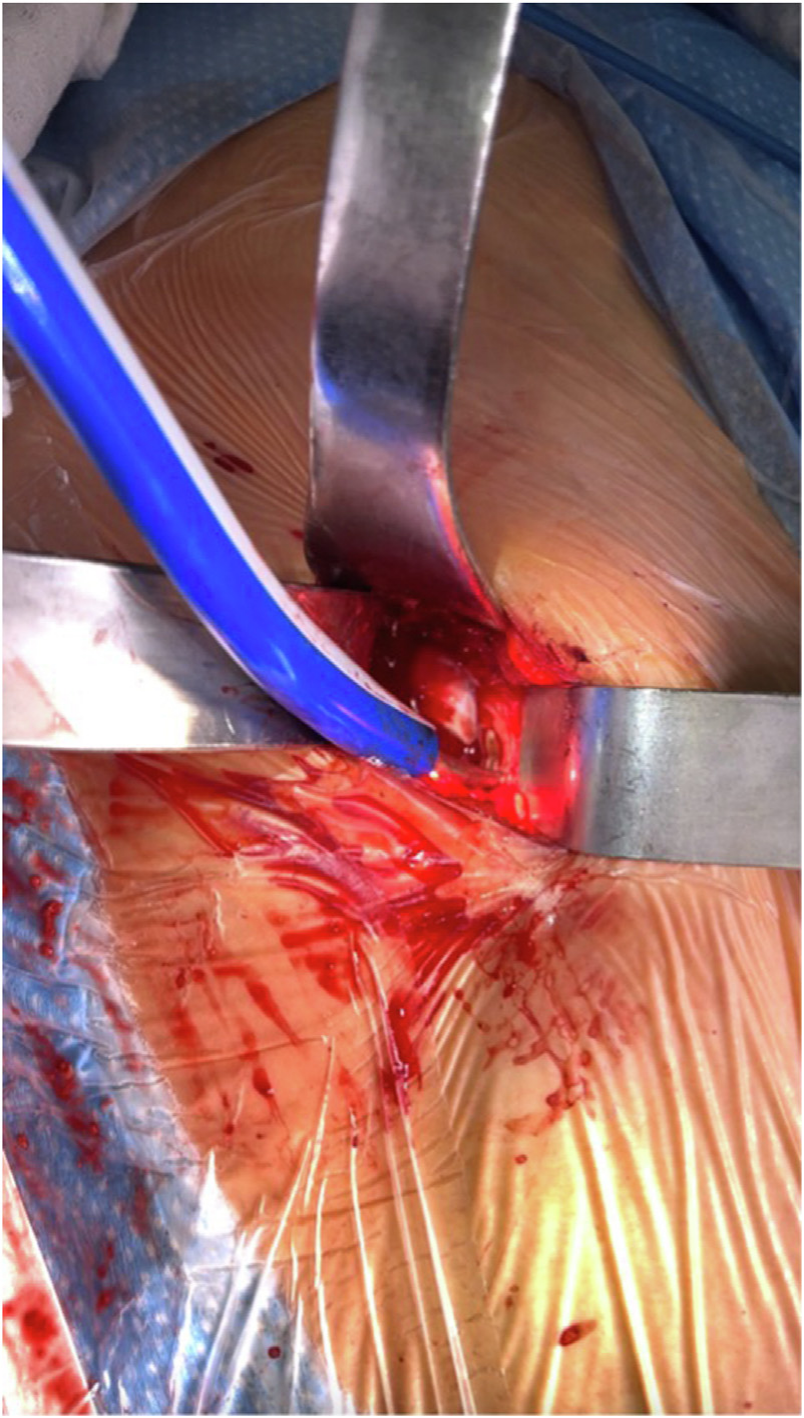
Intraoperative view of the iliopsoas tendon. Cystic lesion on the iliopsoas tendon caused by persistent friction from the femoral head component.

**Figure 8:**
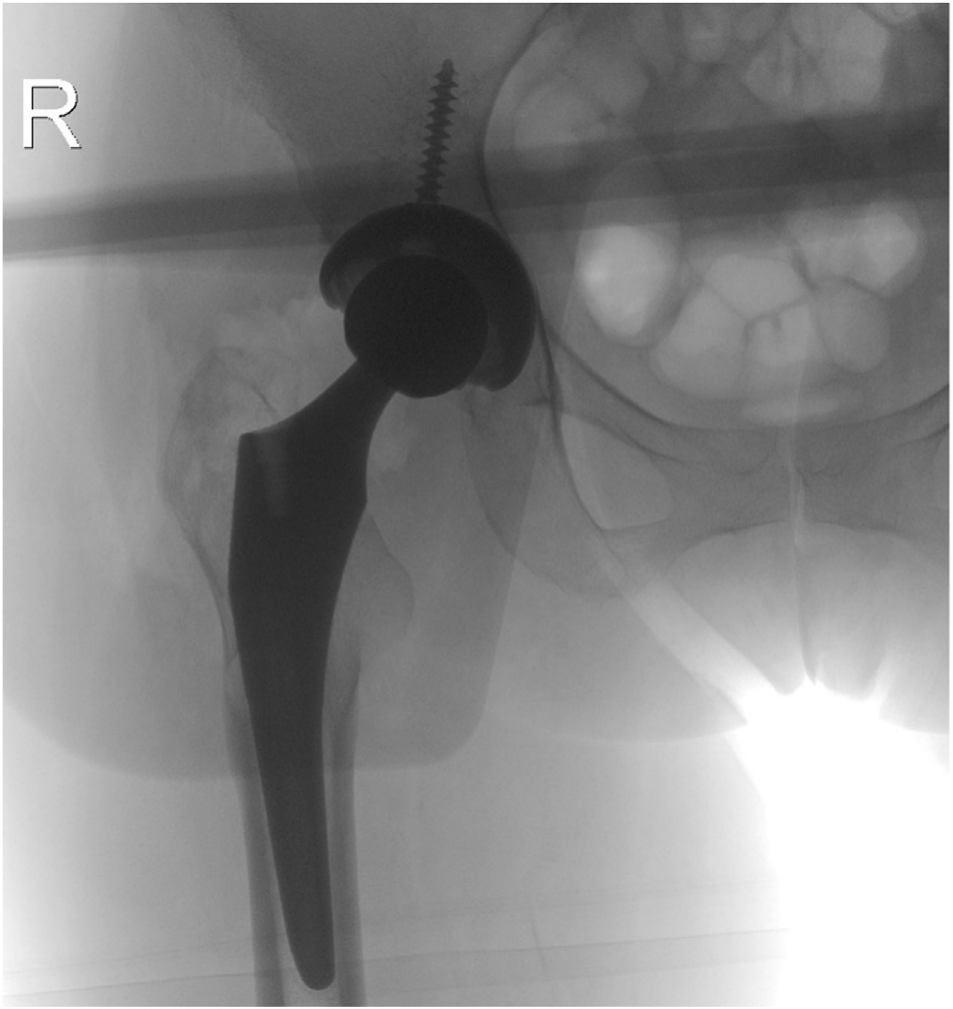
Intraoperative AP view of the right hip from the revision total hip arthroplasty. Radiogram demonstrating a total hip arthroplasty revision with well-aligned components and no signs of fracturing.

In August 2022, the patient underwent a revision of the acetabular component of the right hip with iliopsoas tenotomy via an anterior approach. Intraoperatively, there was significant hemorrhage product inside the tendon and the sheath itself. Approximately 20-25 cc of chronic appearing hematoma was drained from the tendon sheath and sent for culture and pathology. A cystic defect was appreciated in the deep portion of the iliopsoas tendon that was directly overlying the femoral head (Fig. 7). There was no significant wear pattern on the ceramic head, but the acetabular liner had a yellowish hue and was subsequently removed and sent for analysis. A 48-mm reamer was used to medialize the component and adjust the inclination so it sat below and prevented irritation to the anterior structures. A 50-mm Trident cup and 2 screws were placed into the pelvis. The cup was impacted in place onto the internal portion of the acetabulum. The position of the acetabular component was confirmed using AP and lateral fluoroscopy (Fig. 8). A 32-mm Trident head and an anteriorly placed 10-degree elevated liner were placed to give more anteversion to decrease overhang of the acetabular component and limit the risk of dislocation. The leg lengths were clinically and radiographically appropriate. The hip was confirmed stable with deep flexion, extension, and rotation.

**Figure 9:**
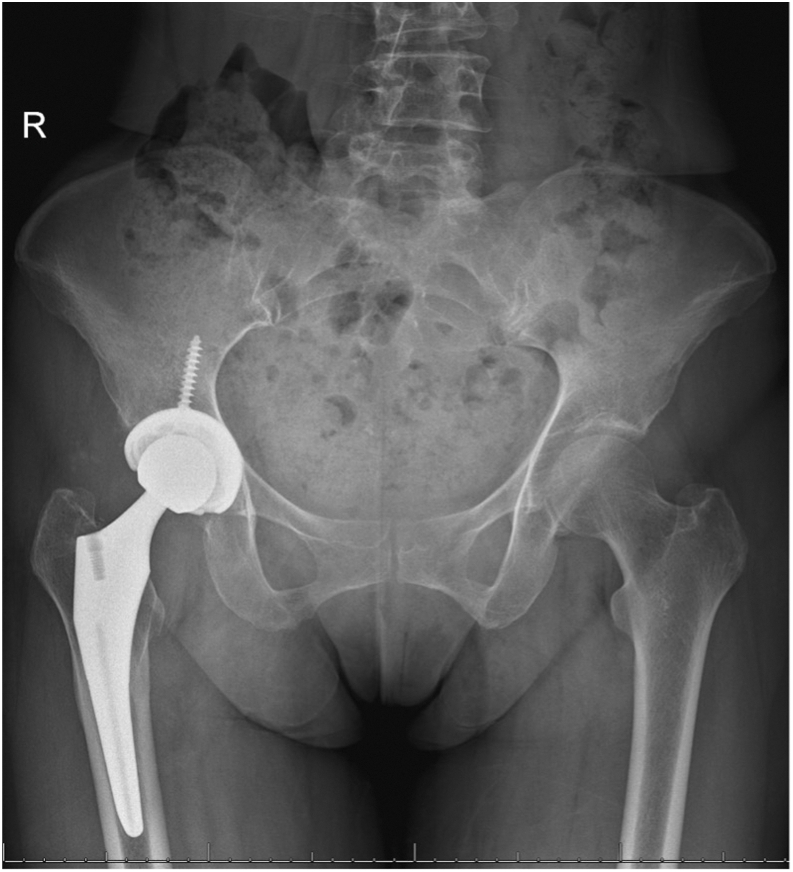
Postoperative AP view of pelvis approximately 1 year after total hip arthroplasty revision. Radiogram demonstrating well-aligned acetabular and femoral components without signs of loosening, fracture subsidence, or dislocation.

Tissue samples from the joint had no growth on culture. Pathology of the hip synovium, including a frozen section, demonstrated reactive fibrosis and hemosiderin deposition consistent with a hematoma and inflammation. The patient was discharged home the day after surgery. Following surgery, the patient had significant pain relief and functional improvement. She was last seen in May 2023, and a physical exam demonstrated full hip active and passive range of motion. Postoperative radiogram were unremarkable (Fig. 9). The patient was contacted in July 2024 and reported that she was very satisfied with the results of her revision surgery and could walk and carry out her daily activities without pain or assistance.

## Discussion

To our knowledge, this is the first report of iliopsoas tendonitis presenting as a pseudotumor secondary to a large intratendon hematoma and cystic tendon lesion following primary THA. Hematomas after arthroplasty of other joints, such as knee and shoulder arthroplasty, have been documented, but there is a lack of literature describing intratendon hematomas in these settings [5,6]. Hematomas of the iliopsoas muscle in native hips are rare but typically occur secondary to trauma or spontaneously in patients with hemorrhagic risk factors, such as hemophilia, chronic kidney disease requiring dialysis, or anticoagulation therapy [7,8]. Iliopsoas muscle hematomas after THA can be a result of blood vessel damage caused by drilling or the placement of a screw in the acetabular component or by impingent due to mispositioning of the acetabular component [9,10]. In this case, the patient was not on anticoagulation therapy, and her symptoms presented without direct trauma after lower body exercise. Although symptom onset was 2.5 years after surgery, THA must be considered a contributing factor. This patient had a sudden onset of pain with reduced mobility after exercise caused by acute bleeding within the iliopsoas muscle and residual limited flexion due to mass effect from hematoma formation. The acute presentation of her intratendon hematoma secondary to squatting is likely a result of chronic iliopsoas irritation from the femoral head.

Postoperative groin pain following THA can be due to a wide variety of pathologies, which can complicate diagnosis and treatment. This case further illustrates the diagnostic complexity of groin pain secondary to iliopsoas pathology in the setting of a THA. The differential diagnosis for groin pain following THA includes infection, fracture, aseptic loosening, heterotopic ossification, reaction to metal, and a variety of intraabdominal, neurological, and vascular pathologies [4]. Therefore, careful attention to the timing of symptom onset, physical examination, and additional investigations, including lab studies and imaging, are critical to accurate diagnosis.

The psoas and iliacus muscles combine to form a strong hip joint flexor while contributing to external rotation [11]. The single tendon inserts into the posteroinferior portion of the lesser trochanter, which is situated posteromedially on the proximal femur [4]. The location of these muscles makes them susceptible to irritation and impingement by the anterior rim of the acetabular component following THA [9]. Therefore, attention must be paid to the anterior overhang of the acetabular component, cup mispositioning, or oversizing of the cup to reduce the chance of iliopsoas tendonitis [12].

Those presenting with iliopsoas tendonitis often present with persistent groin pain with or without a palpable mass. Pain is typically worsened by daily activities such as climbing stairs or standing from a seated position [13]. Pain often follows a period of pain-free mobility, presenting anywhere from 2-96 months following THA [3]. In severe cases, patients with iliopsoas tendinitis find it extremely difficult to lift the symptomatic leg in a seated position but have minimal pain with hip flexion [14]. Associated iliopsoas bursal distention may present as an inguinal or lower abdominal mass [15]. Workup of iliopsoas tendinitis following THA includes radiogram, ultrasound, and MRI. However, image-guided injection of anesthetics and corticosteroids into the iliopsoas tendon is most effective for diagnosis and treatment [16]. Definitive management ranges from physical therapy, non-steroidal anti-inflammatory drugs, and local injections to tendon debridement, tenotomy, or acetabular revision, depending on the severity of the patient’s symptoms [13,16].

The patient, in this case, had a THA with complete recovery, and 2.5 years later, presented with sudden onset pain with an associated palpable groin mass. The patient’s workup included consultation from multiple orthopaedic and general surgeons, CRP, erythrocyte sedimentatin rate, and imaging to rule out infection, fracture, aseptic loosening, and hernias. After careful analysis and extensive conversation with the patient, a shared decision was made to proceed with the revision of the acetabular component of her right hip and iliopsoas tenotomy. The decision to proceed with an acetabular component revision despite normal-appearing radiogram and the lack of an infectious process was mainly due to the patient’s inability to undergo an MRI, and the persistence of symptoms despite fluid aspiration. It was unlikely that iliopsoas tenotomy alone would resolve her symptoms because we believed the muscle would be inflamed and scarred from continuous irritation. Our hypothesis was confirmed intraoperatively. The iliopsoas muscle and sheath were distended from old blood, consistent with chronic hematoma. Persistent friction between the iliopsoas tendon and the femoral head appeared to cause inflammation and friability of the tissue, given the location of the cystic lesion directly overlying the femoral head. It was unlikely that the acetabular screws irritated the iliopsoas, given their posterior-superior placement. The patient’s squatting exercise was the likely inciting event that caused acute intramuscular bleeding. This led to intratendon hematoma formation that compressed the hip joint, causing acute, severe groin pain with radiation into the thigh. Exploration of the hip with acetabular component revision to decrease acetabular cup-to-femoral head ratio, iliopsoas tendon release, and hematoma evacuation eliminated the patient’s pain and improved her mobility.

Buller et al. described that the incidence of anterior iliopsoas impingement and irritation increased as the ratio of cup diameter to native femoral head diameter increased [17]. This patient’s native femoral head was measured to be approximately 47 mm, and she received a 54 mm acetabular cup in the index procedure, giving the patient a cup-to-femoral head ratio of 1.14. Buller et al. further concluded that this increased ratio was significantly associated with anterior iliopsoas impingement and irritation, leading the senior author to downsize the cup in the revision surgery [17].

**Figure 10:**
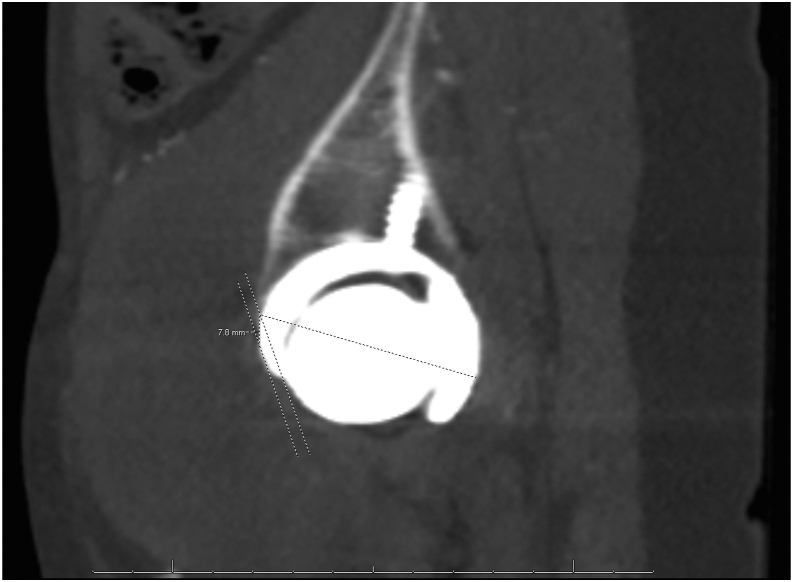
Sagittal view of the right hip. CT scan demonstrating 7.8 mm of anterior acetabular component overhang.

Previous studies, including those by Park et al. and Chalmers et al., have found significant correlations between anterior acetabular overhang, the development of iliopsoas tendinopathy, and the resolution of symptoms after revision. Park et al. found that patients with iliopsoas tendinitis had a higher prevalence of anteroinferior cup prominence ≥8 mm (*P* = .002) [18]. In a retrospective study, Chalmers et al. found that acetabular revision was superior to iliopsoas tenotomy at resolving groin pain in patients with ≥8 mm of prominence (*P* = .07) [16]. Furthermore, Chalmers et al. created a radiographic-based algorithm for groin pain following THA that recommends acetabular revision in patients with acetabular overhang ≥8 mm but only iliopsoas tenotomy for <8 mm [16]. The patient presented in this case had an acetabular overhang measured to be 7.8 mm based on CT scan and still had a resolution of groin pain following acetabular revision (Fig. 10). The algorithm proposed by Chalmers et al. was radiogram-based, making it challenging to apply in this case. Additionally, this patient’s unique presentation and proximity of the measured overhang to 8 mm may be another reason why this patient still saw improvement after revision. Prospective studies using this algorithm and evaluating the implication of CT scans in measuring acetabular overhang are needed to clarify and improve guidelines.

## Summary

This patient’s postoperative course, complicated by iliopsoas tendinitis presenting as an intratendon hematoma with compression features multiple years after a successful THA, is rare. Although postoperative iliopsoas tendonitis is well-established, its presentation varies. Interestingly, anterior prominence of the acetabular component in isolation can cause subacute irritation and inflammation of the iliopsoas tendon, leaving it susceptible to acute injury from trauma or exercise, as seen in this case. Even with an acute on chronic presentation of iliopsoas tenonitis, standard treatment, including acetabular component revision and iliopsoas tendon release, can alleviate symptoms and correct the underlying pathology.

## Conflicts of interest

V.V. Hernandez is a J&J, Zimmer, Enovis consultant, receives research support from OREF, AHHKS, and is a board member of AALOS, AHHKS , AAOS, MOS; all other authors declare no potential conflicts of interest.

For full disclosure statements refer to 10.1016/j.artd.2024.101589

## Informed patient consent

The author(s) confirm that written informed consent has been obtained from the involved patient(s) or if appropriate from the parent, guardian, power of attorney of the involved patient(s); and, they have given approval for this information to be published in this case report (series).

## CRediT authorship contribution statement

**Kevin S. Orton:** Writing – review & editing, Writing – original draft, Conceptualization. **Jonathan M. Stern:** Writing – original draft, Conceptualization. **Natalia Cruz-Ossa:** Writing – review & editing. **Freddy A. Hung:** Writing – review & editing. **Antonio Fernandez-Perez:** Writing – review & editing. **Jean Jose:** Writing – review & editing, Conceptualization. **Victor Hugo Hernandez:** Writing – original draft, Investigation, Conceptualization.

## Appendix A Supplementary data

Conflict of Interest Statement for Fernandez-Perez

Conflict of Interest Statement for Orton

Conflict of Interest Statement for Hung

Conflict of Interest Statement for Jose

Conflict of Interest Statement for Stern

Conflict of Interest Statement for Cruz-Ossa

Conflict of Interest Statement for Hernandez
